# Physicians’ Experiences Using Secure Messaging for Diabetes Management: A Qualitative Study

**DOI:** 10.2196/70816

**Published:** 2025-09-26

**Authors:** Ben Kragen, Maryum Zaidi, Stephanie L Shimada, Ben S Gerber, Cecilia Lozier, Jon A Chilingerian

**Affiliations:** 1 Center for Health Optimization and Implementation Research, VA Bedford Healthcare System, 200 Springs Road (152), Building 70, Bedford, MA, 01730, United States, 1 6032052878; 2 Heller School, Brandeis University, Waltham, MA, United States; 3 Solomont School of Nursing, University of Massachusetts Lowell, Lowell, MA, United States; 4 Department of Population and Quantitative Health Sciences, University of Massachusetts Chan Medical School, Worcester, MA, United States; 5 Division of Endocrine and Diabetes, Baystate Health, Springfield, MA, United States; 6 Public Health and Community Medicine, School of Medicine, Tufts University, Medford, MA, United States

**Keywords:** secure messaging, digital health, patient portals, diabetes, endocrinology, primary care, care coordination

## Abstract

**Background:**

The COVID-19 pandemic led to increased demand for remote management of type 2 diabetes using secure messaging, or patient-provider text-based communication. Prior research on secure messaging has described the content of messages sent for type 2 diabetes management and demonstrated its impact on clinical outcomes. However, there is a gap in knowledge about how secure messaging performs as a communication medium for specific tasks in clinical care (eg, prescription management and discussing medical questions). Additional research is needed to understand physicians’ experiences using secure messaging to communicate with patients about clinical tasks that support diabetes management.

**Objective:**

This study aims to investigate physicians’ experience using secure messaging to communicate with patients about specific clinical tasks for type 2 diabetes management.

**Methods:**

We interviewed a sample of endocrinologists and internists from 2 different medical facilities who have used secure messaging to communicate with adult patients about type 2 diabetes management. Semistructured interviews were used to solicit physicians’ experience using secure messaging for 6 specific tasks that support diabetes management: refilling prescriptions, answering nonurgent medical questions, scheduling appointments, discussing test results, making referral requests, and discussing visit follow-up. Interviews were conducted until we achieved saturation of themes for these tasks. Interview data were collected between 2021 and 2023. Qualitative data were analyzed using the framework method for thematic analysis.

**Results:**

We interviewed 6 internists and 4 endocrinologists (n=10). Physicians reported spending between 2 and 5 hours per day messaging with patients. They observed that secure messaging increased the frequency and timeliness of communication, which improved care coordination and facilitated care delivery between visits. This served as a time-efficient way to iterate specific components of treatment plans, including discussing test results, visit follow-up, scheduling, and prescription refill. Physicians were frustrated with the unstructured nature of secure messages. Patients wrote messages that were often disorganized, confusing, or did not have enough information for the provider to take action. This often made answering nonurgent medical questions difficult. In many cases, poorly structured secure messages resulted in lengthy back-and-forth communications between patients and physicians, which sometimes required a phone call or an office visit to resolve.

**Conclusions:**

Physicians reported that secure messaging supports a longitudinal model of care, where patients can iterate their treatment plan between visits. For tasks with well-defined information boundaries, such as scheduling and prescription refill, physicians reported that secure messaging improved the time efficiency of care delivery. Providers experienced challenges using secure messaging for more complex tasks and often reported not receiving sufficient clinical information. We identified a demand for workflow technologies to process incoming secure messages to improve clarity and ensure that messages have sufficient information to inform decisions on the best course of action.

## Introduction

### Overview

Diabetes has the highest total annual cost of any chronic disease in the United States, with 1 in 4 health care dollars (US $412.9 billion) spent on treating individuals with type 2 diabetes in 2022 [1]. Successful management of type 2 diabetes can be supported by secure messaging, or patient-provider text-based communication through a patient portal [2-11]. Secure messaging offers patients a digital platform to ask providers questions and manage components of their treatment plan between appointments. Patient use of secure messaging has been associated with better diabetes management outcomes [2-9], as well as improved care coordination and communication [9-11]. However, secure messaging has significant limitations in part due to its asynchronous and text-based nature. Studies have shown that secure messaging can lead to miscommunication and delayed or unresolved care [8,12]. This makes secure messaging inappropriate for certain tasks that require immediate action or lengthy communication. In this study, we interviewed physicians to understand their experience using secure messaging as a communication tool to support 6 specific tasks that promote the management of type 2 diabetes: prescription refill, answering nonurgent medical questions, scheduling appointments, discussing test results, making referral requests, and discussing visit follow-up.

### Secure Messaging Increase

Use of secure messaging has increased substantially in recent years, largely spurred by the push to virtual care during the COVID-19 pandemic. During this time, provider adoption of secure messaging was facilitated by changes in billing codes published by the Centers for Medicare & Medicaid Services (CMS) in January 2020 [13]. This was the first time that CMS published incentives for providers and hospitals to engage with secure messaging. Studies indicated that since 2020, patient and/or provider use of secure messaging increased between 50% and 75% across all specialties [14-16], with more than a 50% increase in primary care [17,18]. Looking at provider engagement with secure messaging, a large retrospective study of outpatient messages sent to patients from 2022 to 2023 found that physicians were responsible for the majority of secure messages sent to patients (33%), and that fewer messages came from medical assistants/technicians (26%), nurses (23%), or pharmacists (1%) [19].

### Composition of Secure Messages

Secure messages have been manually coded for themes by teams at the Department of Veterans Affairs [5,6,8,20]. Robinson et al [5,7] and Heisey-Grove and Carretta [20] conducted the only identified studies that analyzed the content of secure messages specifically for diabetes management. Robinson et al [5] found scheduling, referrals, and administrative messages to account for the majority of messages (36%), followed by medication issues (31%), test results (17%), test issues (11%), health issues (3%), and care coordination (1%). In a second study, they found that 91% (293/323) of patients used secure messaging to discuss medication refills, and that 81% (263/323) of patients engaged in at least one thread about referrals, scheduling, or other administrative tasks, while fewer patients used secure messaging to discuss health issues (96/323, 30%) and test results (79/323, 25%) [7]. They found that use of secure messaging by patients with poorly controlled HbA1c largely mirrored trends in the broader sample of patients with type 2 diabetes. Among patients with poorly controlled HbA1C, a majority (161/177, 91%) engaged in medication refill, followed by scheduling/referral/consult (148/177, 84%), medication/equipment issues (117/177, 66%), health issues (60/177, 34%), and test results (47/177, 27%) [7]. Additional research is needed to assess the performance of secure messaging as a communication medium to address specific components of care delivery discussed in the above themes.

### Media Richness Theory

Media richness theory, developed by Daft and Lengel [21] and expanded by Byron [22], is a seminal communication theory that predicts how media (text, video, phone, etc) perform for different types of communication. Media richness theory breaks down communication into a dyadic continuum, ranging from low-complexity to high-complexity communications. Daft and Lengel [21] define low-complexity communication as discussing technical information, such as scheduling, that has well-defined boundaries and little room for ambiguity. High-complexity communication is defined as having poorly defined informational boundaries, greater potential for ambiguity, often requiring iteration, and being necessary for adaptive tasks such as problem-solving.

Media richness theory argues that text-based communications, such as email and secure messaging, are more time-efficient than their “richer” counterparts for low-complexity communications. They argue that a higher-order medium, such as phone or video calls, is overly complex for low-complexity interactions, ultimately creating a time-efficiency mismatch. Two specific categories of secure messaging that fit Daft and Lengel’s [21] description of low-complexity communication are scheduling and prescription management. Both categories gather routine objective data in well-defined situations and are assisted by templates that facilitate information gathering. This informs hypothesis 1: *physicians will report that using secure messaging to accomplish scheduling and prescription management reduces time spent on communication about these tasks*.

Byron [22] argues that text-based mediums such as email and secure messaging are not well suited for high-complexity communications because they do not have the capacity for subtle cues and rapid feedback that is characteristic of phone calls, in-person office visits, and other “richer” mediums. Media richness theory predicts that using text-based mediums for high-complexity communications is less time-efficient compared to using other mediums, such as phone or in-person meetings. The secure messaging categories that meet Daft and Lengel’s [21] definition of high-complexity communications include nonurgent medical questions, discussing test results, visit follow-up, and referral requests. This leads to hypothesis 2: *physicians will report that using secure messaging to discuss nonurgent medical questions, test results, visit follow-up, and referral requests does not reduce provider time spent on communication about these tasks*.

We test these hypotheses using a deductive approach to qualitative analysis, described in the framework method [23]. This allows us to describe the benefits and drawbacks of using secure messaging for specific tasks in the management of type 2 diabetes.

## Methods

### Study Design

This study used a cross-sectional design where semistructured interviews were conducted to solicit physicians’ experience using secure messaging for specific medical tasks in diabetes management. Each physician was interviewed once for approximately 30‐45 minutes. We analyzed this qualitative data using content analysis [24]; data were analyzed by 2 coders through an iterative process using a deductive coding guide and generating inductive codes that emerged during the analysis.

### Study Setting

This study was conducted at 2 health care facilities that serve a catchment of patients in central and western Massachusetts: (1) Baystate Endocrinology in Springfield, Massachusetts, a small outpatient medical center consisting of 9 medical doctors, 4 nurses, and a pharmacist [25]. Baystate Endocrinology is associated with Baystate Health System, a not-for-profit integrated health care system comprising 5 hospitals and over 115 practices [26]. (2) UMass Memorial Medical Center, University Campus, in Worcester, Massachusetts, a large hospital with 1856 clinicians and over 780 staffed beds [27,28]. UMass Memorial Medical Center, University Campus is associated with UMass Memorial Health, an integrated health care system comprising 5 hospitals [29] and over 70 practices [30]. Patients were not charged a copay for secure messaging in either setting. Though CMS billing codes existed for provider reimbursement, these codes were not being used by providers in either setting. Patients receiving care in both settings were able to use secure messaging for the tasks discussed in this paper (ie, prescription refill, discussing nonurgent medical questions, test results, scheduling, referral requests, and visit follow-up).

### Recruitment

Physicians were eligible for recruitment if they were (1) internists or endocrinologists, (2) had adult patients with type 2 diabetes in their panel, (3) spoke English, (4) used secure messaging to communicate with patients about diabetes management, and (5) worked at either Baystate Medical Center Division of Endocrinology or UMass Memorial Medical Center, University Campus. We used snowball sampling to recruit physicians who met the above eligibility criteria. Physicians were contacted up to 3 times by email for recruitment. At the outset of interviews, physicians were notified that their involvement was completely voluntary and that their responses would remain anonymous outside of the research team. Two participants were asked to provide additional feedback on findings once the paper was written.

We used data saturation to define our sample size. To do this, we interviewed physicians until we heard repeated narratives about the use of secure messaging for specific clinical tasks (prescription requests, nonurgent medical questions, test results, scheduling, referral requests, and visit follow-up). Thus, data saturation was defined as the point where interviews stopped illuminating large gaps in knowledge about these clinical tasks (deductive themes) that defined the scope of the study [31]. We achieved data saturation early in the interview process, by the fifth interview, and did 5 additional interviews to confirm that we had identified the major subthemes regarding the challenges and benefits of using secure messaging for the defined clinical tasks.

### Data Collection

Semistructured interviews were conducted with physicians over Zoom (Zoom Video Communications, Inc) videoconferencing app between December 2021 and February 2023. MZ led 3 interviews and BK led 7 interviews. Interviews lasted between 30 and 45 minutes. Interview questions were shared with the participants via email approximately 1 week prior to the meeting. The agenda of the interview was as follows: we began by asking about physicians’ experience using secure messaging to communicate with patients about 6 specific tasks that support diabetes management: visit follow-up, test results, nonurgent medical questions, referral requests, prescription requests, and scheduling requests. These 6 tasks were identified as the deductive themes of interest. This is because these tasks were offered in a dropdown menu as 6 possible subject headers that patients can use to title their secure messages at both UMass Memorial Health and Baystate Health. We then asked physicians about the workflow of messages sent between patients and providers. This section was exploratory and yielded inductive themes.

Interviews were captured on Zoom video recording software and were transcribed using Zoom autogenerated transcripts. Two team members (BK and MZ) edited the autogenerated transcripts for obvious mistakes on the part of the transcription software (eg, autoreplacing “insulin” with “in sullen”), while maintaining a high degree of naturalization [32]. The authors uploaded transcriptions to the 2 qualitative analysis software programs, Atlas.ti 8 (ATLAS.ti Scientific Software Development GmbH) and Nvivo 12 (QSR International).

### Analysis

Data were coded for themes using Atlas.ti [33] and Nvivo 12 [34], then analyzed using the framework method [23]. This involved a combined deductive-inductive approach discussed by Gale et al [23]. We used deductive content analysis to isolate descriptions of how secure messaging performed as a medium for communication about specific clinical tasks (prescription requests, nonurgent medical questions, test results, scheduling, referral requests, and visit follow-up) which were offered to patients as subject headers for secure messages. We used these descriptions to assess whether the data supported hypotheses 1 and 2 described in the “Media Richness Theory” subsection of the “Introduction”. Simultaneously, we used inductive content analysis to highlight additional major themes related to hospital policy that emerged as relevant to physicians’ experience using secure messaging to communicate with patients about diabetes management.

Team members (BK and MZ) developed the code book. Interrater reliability was established by adapting framework-based thematic analysis for semistructured interviews in multidisciplinary teams [23], where initial coding consensus is prioritized over quantitative metrics. Due to the limited number of items coded together and the different backgrounds of coders, no formal reliability coefficient was calculated. The process of establishing interrater reliability involved 2 cycles [35]. During the first cycle of coding, BK and MZ coded the same 2 interviews separately, then met to discuss and achieve consensus on the deductive and inductive themes to develop a working analytic framework. Once interrater reliability was established, remaining transcripts were divided between BK and MZ for a second cycle of content analysis. During this second cycle, these 2 team members had ad hoc communications to discuss additional emergent themes and iterate the analytic framework.

Once all transcripts were coded, BK and MZ each compiled a list of final themes with a definition of each code, interviewer reflections about the code, and 3 to 5 representative quotes for each code. BK and MZ shared the list of codes, definitions, and quotes with all members of the research team, who then met with coders to review and consolidate themes for presentation as results of the study.

### Ensuring Rigor and Trustworthiness

We used established methods to strengthen the rigor and trustworthiness of the data collection, analysis, and reporting. Our interview guide (Multimedia Appendix 1) was informed by theory [21] and co-developed by JAC and BK. The interview guide was then submitted to the Brandeis Institutional Review Board (IRB) for approval. Interviewers BK and MZ used the approved interview guide to conduct semistructured interviews with participants. BK and MZ then used the framework method for the analysis of qualitative data to inform data analysis [23]. The Consolidated Criteria for Reporting Qualitative Research (COREQ) checklist [36] was used to ensure complete and detailed reporting on the study design (Checklist 1).

### Ethical Considerations

This study was determined to be exempt by the IRB for Brandeis University (#22110R-E). Written informed consent was waived by the Brandeis IRB, and verbal consent was collected at the time of the interviews. Interviews took place during working hours, and no compensation was provided to participants. Video files and interview transcripts were stored on an encrypted web platform. Interviews were anonymized at the time of transcription, which were then transferred to Atlas.TI for analysis. BK and MZ had access to the identified data. Data were deidentified before it was shared with the remaining members of the research team.

## Results

### Descriptive Statistics

A total of 10 interviews were conducted with physicians (endocrinologists: n=4, internists: n=6), representing 40% of the 25 physicians who were contacted to participate. Descriptive statistics of the sample are shown in Table 1.

**Table 1.** Sample characteristics (n=10).

Characteristics	Physicians, n (%)
Specialty	
Endocrinology	4 (40)
Internal Medicine	6 (60)
Healthcare facility	
UMass Memorial Medical Center, University Campus	8 (80)
Baystate Endocrinology	2 (20)
Legal sex	
Female	4 (40)
Male	6 (60)
Years in practice	
0‐10	2 (20)
11‐20	3 (30)
20+	5 (50)

### Themes Observed in Interviews

We achieved data saturation early in the interview process, by the fifth interview, and did 5 additional interviews to confirm that we had identified major components in the deductive themes of interest. In addition to deductive themes, qualitative analysis of interviews revealed several notable inductive themes displayed in Textbox 1.

Textbox 1. **Deductive and inductive themes observed in interviews.**

Deductive themes

- Prescription requests
- Nonurgent medical questions
- Test results
- Scheduling
- Referral requests
- Visit follow-up

Inductive themes

- Extra work for the physician
- Lack of knowledge or uncertainty about billing
- Impacts on timeliness of communication
- Expanded record of management

### Deductive Themes

#### Prescription Requests

Secure messaging was widely reported to save time on prescription management. The main time efficiency comes from the fact that secure messaging is well integrated with prescription management workflow templates offered by the 2 hospital systems. Once submitted by patients, prescription refill requests were automatically transferred to staff, who checked the order against the active medication list and ensured that the patient’s lab results were up to date. The prescription management portal displayed the refill protocol as well as gave access to the patient’s electronic health records, providing all the information in one place.

Challenges arose with prescription refill when patients and/or front desk staff missed critical information, such as the preferred pharmacy. Prescribing opioids and other controlled substances further complicated the process and required a phone call from the physician. One physician reported preferring a phone call for all prescriptions because it allowed them to have a conversation with the patient and correct misunderstandings about the medication regimen. Some exemplar quotes regarding prescription requests are as follows:

A physician can authorize five medications with one click if they have been queued up properly.

I think it’s convenient for patients to actually just request their medication refill rather than having to call and wait on the phone. Not everybody’s gonna have the opportunity to be able to ask for prescriptions and things during the day when the office is open, so it becomes easier for them to communicate directly through the portal.

One patient had headaches and was wondering about refilling a prescription from a neurologist. We talked on the phone and found out that the patient was not taking the prescription correctly.

#### Nonurgent Medical Questions

Physicians found that secure messaging was more time-efficient than a phone call or an office visit for a question with well-defined parameters, such as the frequency or dosage of medication. They also spoke about secure messaging, saving patients the hassle of having to wait in a phone queue to talk to a provider, which both improved patient experience and made communication possible for patients with busy schedules.

We heard a repeated theme across physicians that patients’ education level and familiarity with text-based communication moderated their ability to use secure messaging to discuss nonurgent medical questions. Patients’ ability to write concisely and include necessary medical information varied widely. In addition, physicians reported that many patients do not know what physicians need to make a decision or take clinical action. In some cases, medical questions initiated a long back-and-forth exchange that would have been better served by a phone call. Physicians offered suggestions to improve communication, including (1) developing a program to coach physicians and patients about the most effective way to communicate over secure messaging, and (2) modifying the secure messaging template to solicit necessary clinical information. Some exemplar quotes regarding prescription requests are as follows:

I’m so glad to have that ability [to communicate with a patient through secure messaging], because otherwise either they call and they can’t get through and it’s phone tag and it won’t be until I see them in three months or six months or a year that we correct that very straightforward problem. For those types of things, it’s really great.

But if it’s a simple um, for example, there are patients that we see that are on a specific regiment for diabetes. They send. They say, Okay, can you look at my [glucometer] download, or at my continuous glucose? And do you recommend any changes, since it’s not anything urgent. I will just respond to them [at my convenience].

Others are 20 questions that go back and forth where they send one sentence at a time. A lot of people using the portal have not had to communicate electronically for a living.

#### Test Results

Secure messaging gave physicians the ability to quickly add their interpretation to test results, which are automatically shared with patients in accordance with the 21st Century Cures Act. Several physicians noted this legislation posed a challenge; often, patients receive test results before physicians have a chance to interpret results for them, which causes undue anxiety for the patient. Secure messaging facilitated timely interpretation, which improved patient experience. In addition, opening a dialog via secure messaging gave patients the opportunity to ask follow-up questions and coordinate a treatment plan in response to the test result, which could replace an additional follow-up visit.

Patient comprehension was often cited as a reason to use a phone call or office visit instead of secure messaging to explain test results. For more complicated results, such as a magnetic resonance imaging or a computed tomography scan, physicians reported that a phone call was more efficient. One physician talked about preferring phone calls because they facilitated a teach-back method which allowed the physician to gauge patient comprehension. “OK I explained it to you, now explain it back to me.” In other instances, physicians talked about preferring a phone call to explain more sensitive test results, such as a challenging diagnosis, because the phone allowed for more expression of empathy. Some exemplar quotes regarding prescription requests are as follows:

It’s a race to put your comments on a result so patients don’t get anxious and interpret it on their own. Secure messaging helps with this speed. This helps to head off the anxious phone call, or even worse, the anxious phone call to the on-call doctor.

Providers love forwarding test results with interpretation. It’s a huge positive, especially since the Cures Act.

So the patient, when they open up the results, they’ll see my message attached to it, so I... I can either do it that way, or I could send them a separate message, saying, I saw your results of your HbA_1c_ and it was high, and I think you need to increase your dose of metformin.

#### Scheduling

There was consistent agreement among physicians that using secure messaging made scheduling more time-efficient. The benefit of secure messaging for scheduling was discussed by all (10/10) interviewees. Participants reported that most of the scheduling messages were handled by hospital administrative staff. One observed drawback was that patients were often given an appointment several months in the future when they could have gotten an earlier appointment if they had spoken with the administrative staff. A second drawback was that scheduling systems varied by provider group; some provider groups allowed patients to schedule appointments with specialists without first seeing a primary care provider, which resulted in patients attending appointments with providers who were not equipped to solve a particular medical challenge. Some exemplar quotes regarding prescription requests are as follows:

I think scheduling [over secure messaging] is better for offices and for patients because you don’t have to wait for someone to pick up the phone or get the right person on the phone. Scheduling staff don’t have to try to track the patient down. This is important because if people are working 8‐5 and the office is open 8‐5 it will take 8 phone calls to track the patient down to confirm scheduling.

But someone at [HOSPITAL] who’s working with my office. They look at what was scheduled by the patient and make sure the patient scheduled it appropriately. So, for example, I’m a specialist [endocrinologist], and if a patient scheduled an appointment to see me and wrote the reason for the appointment as skin rash … they will call the patient back and say, “You know you didn’t pick the right, doctor.”

#### Referral Requests

Secure messaging facilitated referral requests for issues that physicians already knew about, but it was often not helpful for new issues. In cases where physicians had already seen a patient for a particular medical issue, staff were able to put in the order for a referral and send it over to the physician to review, making the process of referring only take “a few seconds.” Physicians reported that secure messaging posed a challenge for addressing new symptoms. They often did not have enough information about a new symptom to make a decision about the best course of treatment for the patient. In these instances, physicians would either call the patient or have the patient schedule an appointment. Some exemplar quotes regarding prescription requests are as follows:

Patients will say something like “I want to see a specialist” and 95% of the time I just refer them.

Physical therapy is easy. Other specialists are challenging. For example, I need to know what exactly they want to see a neurologist for, and is it a new thing that is going on. Is the problem something that I’ve evaluated before? I want to do certain tests so that when they see the neurologist the preliminary workup has been done. For a lot of those I need to call them and find out what exactly is going on.

... if I look at their numbers [HbA_1c_], and I say, well, maybe you need to see the diabetes educator. I’ll say, are you okay with that?

#### Visit Follow-Up

Physicians reported that secure messaging allowed patients to bring up questions that they forgot to ask during an appointment. This was particularly important when patients received a new prescription and had a question about how to dose the medication. Often, small questions about adjusting a treatment plan would go unasked if it meant that the patient had to book another appointment. In this way, secure messaging lowered the barrier to communication, facilitated timely communication, and often avoided an in-person visit for follow-up. Some exemplar quotes regarding prescription requests are as follows:

For certain things it’s very useful. It’s extremely useful for pictures/images. I had a patient with shoulder pain and I was going to give shoulder exercises but I forgot to give it before he left. I sent the exercises from Epic in a secure message.

Especially if it’s a weekend... if it’s Friday, and I’m messaging somebody, and I don’t want to wait until Monday until somebody [from the office] comes back to look at it. I... I might just say, okay, well send the reply directly to me because it’s easier in that situation.

### Inductive Themes

#### Extra Work for Physicians

All physicians who were interviewed (10/10) reported that their provider group was struggling to manage the additional work created by the increase in secure messaging since the COVID-19 pandemic. Time spent engaging with messages does not factor into physicians’ official volume of work, termed relative value units, and therefore is not acknowledged by their health care system. Instead, physicians discussed being expected to spend time outside of their work schedules responding to messages. We heard that physicians were spending between 2 and 5 hours per day on secure messaging, with 1 to 4 hours happening after-hours, a phenomenon that several physicians referred to as “pajama time.” Several physicians reported that they were unable to keep up with the increased workload and left secure messages unanswered. Some exemplar quotes regarding prescription requests are as follows:

Messaging [since the start of COVID-19 pandemic] does create extra work for the provider, and I think the vast majority of extra work tends to fall in primary care so it’s a big big big primary care issue and, right now I’m positive, I’m young, I’m early in my career I’m OK, but I can see how it would build up… there may be a need for protected time for providers. An additional half day may ultimately be needed for some of those really high volume providers who are getting a lot of messages.

I can just tell you the volume of inbox messages from the patient portal has just gone up dramatically… I spend two to three hours outside of work answering messages and still have another two hours every single day that I don’t get to see. So there are a lot of messages that go unanswered.

At the end of the month physicians get a statement—how many visits they saw, how many RVUs they billed. This shows up on their report card. One of my missions in life that I keep telling my chairperson about is to measure this new type of work [secure messaging]. This is hurting primary care providers.

#### Lack of Knowledge or Uncertainty About Billing

None of the interviewed physicians (0/10) reported billing for secure messaging. When asked about the CMS billing codes for secure messaging, physicians generally had 1 of 2 responses: they either did not know about the existence of billing codes or said that the current billing scheme was clunky and that it was not worth the small amount of money per message.

Several physicians talked about how the billing structure incentivized them to turn conversations into phone calls or in-person visits that are more easily measured and rewarded. This happened even when physicians thought that secure messaging might provide a better, more timely, or more efficient way to communicate. Some exemplar quotes regarding prescription requests are as follows:

Unlike lawyers, we do not get to bill by time. If someone asks a complex question, it’s effectively the same as them coming into the office. We have the ability to charge for that, but we have not implemented it yet. It is on our roadmap.

I don’t have a solution [for billing for secure messaging], but I think that the first step is to measure the amount of effort.

Yeah, I have not billed for any secure messages. I think we’re supposed to be documenting it as a phone encounter.

Phone calls have been able to be billed for years and providers are not billing for them because it’s a small amount of money and it’s not worth the time to click around.

#### Timeliness of Communication

Several physicians reported that secure messaging greatly improved the longitudinal model of care delivery, or care that extends beyond scheduled encounters. Secure messaging gave patients the opportunity to ask questions that otherwise would have been forgotten and would not come up at the next appointment, which was especially important for physicians who scheduled weeks or months out. The asynchronous nature of secure messaging further supported timely communication because physicians could respond to messages at a time that was convenient for them. This gave physicians flexibility to send communications to patients after business hours, when they would otherwise not be comfortable calling.

In other instances, secure messaging was found to increase the time needed to communicate about a challenge. They talked about disjointed message conversations that went back and forth over several days.

In terms of reaching patients, physicians found that secure messaging was sometimes much faster, and other times unreliable. We saw variation in providers’ ability to view patients’ “read receipts,” when a patient saw a message. Some physicians talked about a function in the portal that alerted them when patients had not opened emails within 72 hours of receiving a message. Others said that they did not know how to view the “read receipt,” which caused them to frequently feel obligated to follow up the message with a phone call. Some exemplar quotes regarding prescription requests are as follows:

This really advances the whole longitudinal fluid kind of model of medicine. Before secure messaging, providers would perform a procedure, and then not see the patient for six months. Now care is extended; the services are more fluid.

It [secure messaging] helps patients feel more connected, heard, and secure.

So I mean they I... I personally find it easier. Some people find it harder. Um. The thing I like about secure messaging is that it’s asynchronous. So they are… asking their question on their time. I’m answering the question on my time. Uh, and we don’t have to sync up our schedules to have that conversation.

#### Expanded Record of Management

Physicians reported that a unique strength of secure messaging was that it created a digital paper trail of conversations and communications. Four providers observed that secure messaging made it possible to connect their notes to pictures and other email attachments, such as a log of blood sugars, lab results, or a readout from a remote patient monitoring device. Connecting text to these images helped physicians to communicate the story of the treatment to patients. Similarly, secure messaging facilitated sending brochures and other content that was translated into several languages in addition to English, which increases equity of care delivery.

Secure messaging facilitates sending email attachments [log of blood sugars as a picture/screenshot, or readouts from aglucometer] which increases adherence. This allows for more timely coordination around getting patients to diabetes control.

[I do not need to]… rely on my memory to figure out what happened in between [visits]. This is all in the chart. I can Just look at all my visits and all my messages and all my communications with the patient.

## Discussion

### Secure Messaging for Low- and High-Complexity Communications

We used qualitative interviews to investigate physicians’ experiences communicating with patients about type 2 diabetes management using secure messaging. For low-complexity communications (scheduling and prescription management), we found that secure messaging was effective and efficient in most of the scenarios. Both facilities offered scheduling and prescription order templates that solicited the necessary information for physicians to make a decision or complete a request. This is a common best practice for hospital systems [37].

Findings were mixed about the use of secure messaging for high-complexity communications (nonurgent medical questions, visit follow-up, discussing test results, and referral requests). The chief advantage over in-person, video, or phone encounters was that secure messaging facilitated rapid communication; the asynchronous nature of the medium allowed both parties to bypass the barrier of scheduling a future encounter to discuss medical questions, test results, or other follow-up topics. This builds on findings from Wade-Vuturo et al [38] that secure messaging improves the longitudinal model of care. Advantages include better continuity with providers and more efficient, time-limited patient-physician interactions. Secure messaging further created a record of a conversation that was visible to patients and physicians. This allows for better clarity, coordination, and understanding of the treatment plan and visit follow-up by both the patient, physician, and primary care team [10-12]. The centralized email thread further allowed for the sharing of lab results and imaging with attached annotations that highlighted and explained specific findings. For example, a physician could attach notes directly to lab results to explain findings to a patient. This use of multimedia (image+text) adds nuance to Daft and Lengel’s [21] position and supports the notion that communication media are mutually reinforcing, rather than hierarchical, as explained in media richness theory [38,39]. This concept of high and low richness mediums being mutually reinforcing is explored in other frameworks such as media synchronicity theory and relational coordination theory [40,41]. In this project, we found that the use of secure messaging for multimedia communication varied widely by physician comfort with the medium. At one extreme, physicians reported using secure messaging to send up to 4 different types of information, including test results, images, informational brochures, and readouts from remote patient monitoring devices. At the other extreme, a physician reported not knowing how to use secure messaging to share documents with patients.

Our findings agreed with prior research that secure messaging for high-complexity communication made it possible to iterate treatments between appointments [10,11,38]. Improved timeliness may directly address clinical inertia that impedes diabetes management [42,43]. However, in many cases, the advantage of timeliness came at the cost of inefficient use of clinician time when the patient asked multiple questions, asked a confusing question, and/or did not include all the information the physician needed to make a decision. This resulted in lengthy back-and-forth communications that physicians often turned into phone calls or office visits for clarification. This is consistent with the predictions of Daft and Lengel [21] and supports the findings of Lee [12]. In these instances, the conversation was often duplicated in the second meeting, making secure messaging redundant. Future work is needed to identify strategies to avoid this repeated work (eg, prompts for patients, or use of natural language processing to identify missing information) [44].

Physicians in our study perceived that the capacity for secure messaging to support high-complexity communications varies across patients. Some patients appear able to use text to communicate challenging concepts, while others do not. This agrees with critics of media richness theory, who argue that it is not the medium itself, but instead an individual’s familiarity with the medium that predicts its ability to support high-complexity communications [22,45-48]. In this way, individual characteristics such as the patient’s health literacy or familiarity with email could potentially moderate the success of secure messaging as a medium for complex communications; this could widen health disparities if more vulnerable patients have a higher likelihood of secure message exchanges that fail to successfully result in an appropriate health care action. There is a need for mechanisms that standardize expectations about the content of secure messages to support clear and concise communication. Some possible technologies include a prompt for patients that requests key information, voice entry for those who cannot type easily, and the use of artificial intelligence to review patient messages and suggest additional information to include.

### Practice Implications: Opportunities to Structure Incoming Secure Messages

#### Develop Message Prompts

Physicians are frustrated by the lack of structure in secure messages. It may be helpful to supplement an open-ended secure message with prompts at the top of the open-ended textbox to ask the patient to include specific information in their message [1]. Prompts could either be kept broad or developed as a list of information needed for specific tasks, such as discussing a prescription, discussing test results, or following up after a visit. This would help to communicate expectations about information to include in the message. Specific prompts have been successful in the context of e-visits for referral requests, or messages sent between primary care providers and specialists [49,50].

#### Develop Workflows to Screen Information

Physicians observed that secure messages were often processed by administrative staff, technicians, and/or nurses, and that their engagement was highly varied. For example, one physician described a front desk staff member who would screen messages for information gaps and reach out to patients for additional information. Other front desk staff would not feel comfortable interceding and responding to the patient. Instead, they would forward messages directly to physicians. Developing consistent guidelines/training for administrative staff to read messages, summarize key information, and follow up with patients for additional information may help to reduce physician burden. Workflows for messaging triaging and message summary can be further aided by natural language processing and other machine learning methods that process information [51]. Together, these strategies have the potential to help reduce the burden on physicians, which in turn will reduce burnout and the overall cost of care. However, the quality of care and propensity for medical errors should be studied in this context prior to widespread implementation.

### Practice Implications: Opportunities to Facilitate Outgoing Secure Messages

#### Train Providers on Best Practices in Secure Message Use

We found that some physicians do not consistently use secure messaging for 2 main reasons: (1) lack of best practices about how to use the medium and (2) lack of incentives. There is a well-documented absence of best practices for the use of secure messaging [52-55]. Training focused on the process of secure messaging would help providers to maximize the benefits of messaging and avoid repeated tasks. For example, teaching providers to use read receipts would avoid a follow-up phone call to see if a patient received a message. Similarly, teaching physicians to connect messages to lab results and/or imaging would avoid the need to retype test results in the message.

#### Give Providers Time and Credit for Secure Messaging

Physicians in this study observed that there was (1) no widely-used internal metrics to track their use of secure messaging and (2) no dedicated time to manage their inboxes. Instead, physicians were expected to use downtime between appointments or stay late to complete this work. This highlights a need for practice-level strategies to acknowledge secure messaging-related tasks. One approach to record this work is to educate providers about the billing codes established by CMS [13]. However, providing incentives without changes to workload is not sufficient and can widen inequality in health care delivery. Prior work has found that federal incentives for secure messaging were associated with increased secure messaging in high-resource settings, but no change in low-resource settings [56]. Providers serving underresourced communities may be too busy or lack adequate knowledge or resources to engage with messaging [56]. At the patient level, billing for secure messaging has been associated with a decrease in message use by underserved populations, including patients who are Latinx and/or self-insured [14]. Charging patients a copay for secure messaging may limit engagement among low socioeconomic groups and exacerbate existing disparities in access. Additional work is needed to develop a mechanism to acknowledge provider work without adding financial burden to patients.

### Limitations

There were several limitations to note. First, our study had a limited sample size. We achieved thematic saturation and were hearing the deductive themes highlighted in this paper repeated by the fifth interview. However, the inductive themes discussed in this paper did not reach saturation, as they were not the primary focus of the paper. It would have been helpful to interview additional endocrinologists and primary care providers to improve the transferability of and confidence in our findings. Moreover, this research is limited to the case of diabetes management in 2 hospital settings in Massachusetts. Additional research is needed to understand how secure messaging performs for clinical tasks in other regions and among other clinical subspecialties. Further, the study is prone to response bias as several providers chose not to be interviewed. A last limitation is that the semistructured interviews conducted for this study were exploratory in nature. While they were successful in uncovering the breadth of challenges that physicians faced when they used secure messaging for specific tasks, this approach did not make it possible to infer the percent of physicians who experienced these challenges. Future survey work is needed to investigate the generalizability of our findings among a larger, randomly selected cohort of physicians.

### Conclusions

This study investigates physicians’ experience using secure messaging to communicate with patients about specific tasks for type 2 diabetes management. Physicians felt that secure messaging provided timely and efficient care to patients with type 2 diabetes, and that this medium supported both high- and low-complexity communications. However, messages that were confusing or disorganized often resulted in decreased efficiency of communication with patients as compared to other mediums, such as phone or video. While this study provides insights for virtual health and health care redesign, additional information is required to develop best practice guidelines for secure messaging and to optimize the organizational resources allocated for its implementation. Future work is needed to investigate interventions to improve the organization and clarity of messages (ie, messaging prompts, or the use of artificial intelligence to help patients fill in gaps in content).

## Supplementary material

10.2196/70816 Multimedia Appendix 1 Interview guide.

10.2196/70816 Checklist COREQ checklist.

## Abbreviations

CMS: Centers for Medicare & Medicaid Services
COREQ: Consolidated Criteria for Reporting Qualitative Research
IRB: institutional review board

## References

[1] Parker ED, Lin J, Mahoney T (2024). Economic costs of diabetes in the U.S. in 2022. Diabetes Care.

[2] Chung S, Panattoni L, Chi J, Palaniappan L (2017). Can secure patient-provider messaging improve diabetes care?. Diabetes Care.

[3] Harris LT, Haneuse SJ, Martin DP, Ralston JD (2009). Diabetes quality of care and outpatient utilization associated with electronic patient-provider messaging: a cross-sectional analysis. Diabetes Care.

[4] Harris LT, Koepsell TD, Haneuse SJ, Martin DP, Ralston JD (2013). Glycemic control associated with secure patient-provider messaging within a shared electronic medical record: a longitudinal analysis. Diabetes Care.

[5] Robinson SA, Zocchi MS, Netherton D (2020). Secure messaging, diabetes self-management, and the importance of patient autonomy: a mixed methods study. J Gen Intern Med.

[6] Robinson SA, Netherton D, Zocchi M, Purington C, Ash AS, Shimada SL (2021). Differences in secure messaging, self-management, and glycemic control between rural and urban patients: secondary data analysis. JMIR Diabetes.

[7] Robinson SA, Zocchi M, Purington C (2023). Secure messaging for diabetes management: content analysis. JMIR Diabetes.

[8] Shimada SL, Petrakis BA, Rothendler JA (2017). An analysis of patient-provider secure messaging at two Veterans Health Administration medical centers: message content and resolution through secure messaging. J Am Med Inform Assoc.

[9] Shimada SL, Zocchi MS, Hogan TP (2020). Impact of patient-clinical team secure messaging on communication patterns and patient experience: randomized encouragement design trial. J Med Internet Res.

[10] Eschler J, Liu LS, Vizer LM (2015). Designing asynchronous communication tools for optimization of patient-clinician coordination. AMIA Annu Symp Proc.

[11] Hefner JL, MacEwan SR, Biltz A, Sieck CJ (2019). Patient portal messaging for care coordination: a qualitative study of perspectives of experienced users with chronic conditions. BMC Fam Pract.

[12] Lee JL, Matthias MS, Huffman M, Frankel RM, Weiner M (2020). Insecure messaging: how clinicians approach potentially problematic messages from patients. JAMIA Open.

[13] (2020). Medicare telemedicine health care provider fact sheet. CMS.

[14] Holmgren AJ, Downing NL, Tang M, Sharp C, Longhurst C, Huckman RS (2022). Assessing the impact of the COVID-19 pandemic on clinician ambulatory electronic health record use. J Am Med Inform Assoc.

[15] Meyer C, Sherman JF, Sheinberg M (2020). How has COVID-19 affected patient access to health records at Penn Medicine LG Health?. J Lanc Gen Hosp.

[16] Saleem JJ, Read JM, Loehr BM (2020). Veterans’ response to an automated text messaging protocol during the COVID-19 pandemic. J Am Med Inform Assoc.

[17] Haun JN, Panaite V, Cotner BA (2022). Primary care virtual resource use prior and post COVID-19 pandemic onset. BMC Health Serv Res.

[18] Spelman JF, Brienza R, Walsh RF (2020). A model for rapid transition to virtual care, VA Connecticut primary care response to COVID-19. J Gen Intern Med.

[19] Baratta LR, Harford D, Sinsky CA, Kannampallil T, Lou SS (2023). Characterizing the patterns of electronic health record-integrated secure messaging use: cross-sectional study. J Med Internet Res.

[20] Heisey-Grove DM, Carretta HJ (2020). Disparities in secure messaging uptake between patients and physicians: longitudinal analysis of two national cross-sectional surveys. J Med Internet Res.

[21] Daft RL, Lengel RH (1986). Organizational information requirements, media richness and structural design. Manage Sci.

[22] Byron K (2008). Carrying too heavy a load? The communication and miscommunication of emotion by email. Acad Manag Rev.

[23] Gale NK, Heath G, Cameron E, Rashid S, Redwood S (2013). Using the framework method for the analysis of qualitative data in multi-disciplinary health research. BMC Med Res Methodol.

[24] Sandelowski M, Barroso J (2006). Handbook for Synthesizing Qualitative Research.

[25] Baystate endocrinology - Springfield. Baystate Health.

[26] About Baystate Health. Baystate Health.

[27] Find healthcare providers: compare care near you. Medicare.

[28] UMass Memorial Medical Center - university campus. American Hospital Directory.

[29] About us. UMass Memorial Health.

[30] UMass Memorial Medical Group. UMass Memorial Health.

[31] Saunders B, Sim J, Kingstone T (2018). Saturation in qualitative research: exploring its conceptualization and operationalization. Qual Quant.

[32] Oliver DG, Serovich JM, Mason TL (2005). Constraints and opportunities with interview transcription: towards reflection in qualitative research. Soc Forces.

[33] ATLAS.ti for Mac & Windows. ATLAS.ti.

[34] About NVivo. NVivo.

[35] Saldana J (2021). The Coding Manual for Qualitative Researchers.

[36] Consolidated Criteria for Reporting Qualitative Research (COREQ): a 32-item checklist for interviews and focus groups. EQUATOR Network.

[37] Zhao P, Yoo I, Lavoie J, Lavoie BJ, Simoes E (2017). Web-based medical appointment systems: a systematic review. J Med Internet Res.

[38] Wade-Vuturo AE, Mayberry LS, Osborn CY (2013). Secure messaging and diabetes management: experiences and perspectives of patient portal users. J Am Med Inform Assoc.

[39] Hajjar L, Kragen B (2021). Timely communication through telehealth: added value for a caregiver during COVID-19. Front Public Health.

[40] Claggett JL, Karahanna E (2018). Unpacking the structure of coordination mechanisms and the role of relational coordination in an era of digitally mediated work processes. Acad Manag Rev.

[41] Hoffer Gittell J (2002). Coordinating mechanisms in care provider groups: relational coordination as a mediator and input uncertainty as a moderator of performance effects. Manage Sci.

[42] Gabbay RA, Kendall D, Beebe C (2020). Addressing therapeutic inertia in 2020 and beyond: a 3-year initiative of the American Diabetes Association. Clin Diabetes.

[43] Phillips LS, Branch WT, Cook CB (2001). Clinical inertia. Ann Intern Med.

[44] Ayers JW, Poliak A, Dredze M (2023). Comparing physician and artificial intelligence chatbot responses to patient questions posted to a public social media forum. JAMA Intern Med.

[45] Dennis AR, Fuller RM, Valacich JS (2008). Media, tasks, and communication processes: a theory of media synchronicity. MIS Q.

[46] Fulk J, Steinfield CW, Schmitz J, Power JG (1987). A social information processing model of media use in organizations. Communic Res.

[47] Ishii K, Lyons MM, Carr SA (2019). Revisiting media richness theory for today and future. Human Behav and Emerg Tech.

[48] Ishii K (2005). The human side of the digital divide: media experience as the border of communication satisfaction with email. J Tech Writ Commun.

[49] Battaglia C, Lambert-Kerzner A, Aron DC (2015). Evaluation of e-consults in the VHA: provider perspectives. Fed Pract.

[50] Savoy A, Militello L, Diiulio J (2019). Cognitive requirements for primary care providers during the referral process: information needed from and interactions with an electronic health record system. Int J Med Inform.

[51] De A, Huang M, Feng T, Yue X, Yao L (2021). Analyzing patient secure messages using a Fast Health Care Interoperability Resources (FIHR)-based data model: development and topic modeling study. J Med Internet Res.

[52] Alanazi A, Anazi YA (2019). The challenges in personal health record adoption. J Healthc Manag.

[53] Arvisais-Anhalt S, Wickenhauser KA, Lusk K, Lehmann CU, McCormack JL, Feterik K (2022). Direct secure messaging in practice-recommendations for improvements. Appl Clin Inform.

[54] Hefner JL, Sieck CJ, Walker DM (2022). Patient and physician perspectives on training to improve communication through secure messaging: clarifying the rules of engagement. Health Care Manage Rev.

[55] Sieck CJ, Hefner JL, Schnierle J (2017). The rules of engagement: perspectives on secure messaging from experienced ambulatory patient portal users. JMIR Med Inform.

[56] Senft N, Butler E, Everson J (2019). Growing disparities in patient-provider messaging: trend analysis before and after supportive policy. J Med Internet Res.

