# Supplementary material for: Physicians’ Experiences Using Secure Messaging for Diabetes Management: A Qualitative Study
Source: JMIR Diabetes. 2025 Sep 26;10:e70816. doi: 10.2196/70816 (PMC12468167; doi:10.2196/70816)
Supplement: Multimedia Appendix 1 [file diabetes-v10-e70816-s001.docx]

**Interview Guide**

Physicians’ Experience Using Secure Messaging for Diabetes Management

“We are hoping to use the information collected from this interview to inform health care policy about secure messaging. The information that you provide will be kept anonymous and your answers will not be shared with anyone outside of this research project. If we wish to use a quote from your interview we will reach out to make sure that we are representing you as you wish to be represented. If you would like to sign a paper consent form, we can provide that for you. Otherwise your verbal consent is sufficient. If you would like to skip a question or stop the interview at any time just let me know. Do you have any questions?”

● Have you used secure messaging to **fill prescriptions for diabetes medication**?

○ If yes- can you talk about how using secure messaging made this task easier or harder?

● Have you used secure messaging to **answer medical questions about diabetes management**?

○ If yes- can you talk about how using secure messaging made this task easier or harder?

● Have you used secure messaging to **schedule appointments for diabetes management?**

○ If yes- can you talk about how using secure messaging made this task easier or harder?

● Have you used secure messaging to **discuss test results for diabetes management**?

○ If yes- can you talk about how using secure messaging made this task easier or harder?

● Have you used secure messaging to **make referral requests for diabetes management**?

○ If yes- can you talk about how using secure messaging made this task easier or harder?
