# Supplementary material for: Physicians’ Experiences Using Secure Messaging for Diabetes Management: A Qualitative Study
Source: JMIR Diabetes. 2025 Sep 26;10:e70816. doi: 10.2196/70816 (PMC12468167; doi:10.2196/70816)
Supplement: Checklist [file diabetes-v10-e70816-s002.docx]

| **No** | **Item** | **Guide questions/description** | **Authors comments** |
| --- | --- | --- | --- |
| **Domain 1: Research team and reflexivity** |  |  |  |
| Personal Characteristics |  |  |  |
| 1. | Interviewer/facilitator | Which author/s conducted the interview or focus group? | Interviews were conducted by:  Dr. Ben Kragen and Dr. Maryum Zaidi |
| 2. | Credentials | What were the researcher's credentials? *E.g. PhD, MD* | Ben Kragen, PhD, MBA  Maryum Zaidi, PhD, RN  Ben S Gerber, MD, MPH  Stephanie L Shimada, PhD  Cecilia Lozier, MD  Jon A Chilingerian, PhD |
| 3. | Occupation | What was their occupation at the time of the study? | Ben Kragen: Post-Doctoral Research Fellow, U.S. Department of Veterans Affairs  Maryum Zaidi: Assistant Professor, University of Massachusetts, Lowell  Ben S Gerber: Professor, University of Massachusetts, Chan Medical School  Stephanie L Shimada: Principal Investigator, U.S. Department of Veterans Affairs  Cecilia Lozier: Chief, Department of Medicine, Baystate Health  Jon A Chilingerian: Professor, Brandeis University |
| 4. | Gender | Was the researcher male or female? | Ben Kragen: Male  Maryum Zaidi: Female  Ben S Gerber: Male  Stephanie L Shimada: Female  Cecilia Lozier: Female  Jon A Chilingerian: Male |
| 5. | Experience and training | What experience or training did the researcher have? | BK, MZ, BG, SS, and JC had a minimum of 3 years of health services research experience at the time of the study.  BG, and CL had a minimum of 5 years of clinical experience at the time of the study. |
| Relationship with participants |  |  |  |
| 6. | Relationship established | Was a relationship established prior to study commencement? | Relationship with research participants was established prior to study commencement. Informal recruitment of research participants was conducted using professional networks. BG, CL, and MZ participated in asking colleagues to participate in interviews. |
| 7. | Participant knowledge of the interviewer | What did the participants know about the researcher? e*.g. personal goals, reasons for doing the research* | Participants knew about the researchers’ interests in learning about how to best leverage secure messaging for diabetes management, as was communicated in the outreach email. |
| 8. | Interviewer characteristics | What characteristics were reported about the interviewer/facilitator? e.g. *Bias, assumptions, reasons and interests in the research topic* | Participants knew about researchers’ interest in the topic of secure messaging and diabetes management. |
| **Domain 2: study design** |  |  |  |
| Theoretical framework |  |  |  |
| 9. | Methodological orientation and Theory | What methodological orientation was stated to underpin the study? *e.g. grounded theory, discourse analysis, ethnography, phenomenology, content analysis* | We used the framework method to analyze transcripts generated from semi-structured interviews. This was helpful in assessing a priori assumptions – informed by media richness theory - that the authors had about the specific use cases for secure messaging. BK and MZ coded transcripts using content analysis, then developed a list of deductive themes (informed by media richness theory) and inductive themes that surfaced form the research. |
| Participant selection |  |  |  |
| 10. | Sampling | How were participants selected? *e.g. purposive, convenience, consecutive, snowball* | Snowball sampling was used to select participants at two medical centers. |
| 11. | Method of approach | How were participants approached? e*.g. face-to-face, telephone, mail, email* | Participants were approached via email. |
| 12. | Sample size | How many participants were in the study? | n = 10 |
| 13. | Non-participation | How many people refused to participate or dropped out? Reasons? | 25 |
| Setting |  |  |  |
| 14. | Setting of data collection | Where was the data collected? e*.g. home, clinic, workplace* | Data was collected via Zoom. |
| 15. | Presence of non-participants | Was anyone else present besides the participants and researchers? | Nobody else was present aside from the participants and the researchers. |
| 16. | Description of sample | What are the important characteristics of the sample? *e.g. demographic data, date* | **Medical specialty:** 4 endocrinologists, 6 internists  **Hospital Network**: 8 from UMass Memorial Medical Center, 2 from Baystate Medical Center  **Legal sex of participants**: 6 male, 4 female  **Years in practice:** 2 (0-10 years), 3 (11-20 years), 5 (20+) |
| Data collection |  |  |  |
| 17. | Interview guide | Were questions, prompts, guides provided by the authors? Was it pilot tested? | Interview questions were provided to participants prior to interviews.  This study was pilot tested among the research team. |
| 18. | Repeat interviews | Were repeat interviews carried out? If yes, how many? | Interviews were not repeated with individuals. |
| 19. | Audio/visual recording | Did the research use audio or visual recording to collect the data? | The research used Zoom recording capabilities, and recorded both audio and visual components. |
| 20. | Field notes | Were field notes made during and/or after the interview or focus group? | Field notes were made during and after the interviews. |
| 21. | Duration | What was the duration of the interviews or focus group? | Interviews lasted approximately 30-45 minutes |
| 22. | Data saturation | Was data saturation discussed? | Data saturation was used to define the sample size. We interviewed physicians until we heard repeated narratives about deductive themes. Data saturation was achieved by the fifth interview, then another five interviews were conducted to confirm. |
| 23. | Transcripts returned | Were transcripts returned to participants for comment and/or correction? | Transcripts were not returned to participants. |
| **Domain 3: analysis and findings** |  |  |  |
| Data analysis |  |  |  |
| 24. | Number of data coders | How many data coders coded the data? | Two coders were used (BK and MZ) |
| 25. | Description of the coding tree | Did authors provide a description of the coding tree? | A description of the coding process was discussed in the methods section under the analysis subheading. |
| 26. | Derivation of themes | Were themes identified in advance or derived from the data? | Six deductive themes were identified in advance, and an additional four inductive themes were derived from the data. |
| 27. | Software | What software, if applicable, was used to manage the data? | Atlas.ti and Nvivo 12 were used to code/manage the data. |
| 28. | Participant checking | Did participants provide feedback on the findings? | Two participants were asked to provide feedback on the findings and were included as co-authors on the paper. |
| Reporting |  |  |  |
| 29. | Quotations presented | Were participant quotations presented to illustrate the themes / findings? Was each quotation identified? e*.g. participant number* | Quotations were presented to illustrate the themes. Quotations were not identified using a participant number. |
| 30. | Data and findings consistent | Was there consistency between the data presented and the findings? | Yes |
| 31. | Clarity of major themes | Were major themes clearly presented in the findings? | Yes |
| 32. | Clarity of minor themes | Is there a description of diverse cases or discussion of minor themes? | Additional themes that arose during the coding process were discussed in the results section titled “inductive themes”. |
